# Recombinant human SIRT1 protects against nutrient deprivation-induced mitochondrial apoptosis through autophagy induction in human intervertebral disc nucleus pulposus cells

**DOI:** 10.1186/s13075-015-0763-6

**Published:** 2015-09-15

**Authors:** Shingo Miyazaki, Kenichiro Kakutani, Takashi Yurube, Koichiro Maeno, Toru Takada, Zhongying Zhang, Takuto Kurakawa, Yoshiki Terashima, Masaaki Ito, Takeshi Ueha, Takehiko Matsushita, Ryosuke Kuroda, Masahiro Kurosaka, Kotaro Nishida

**Affiliations:** Department of Orthopaedic Surgery, Kobe University Graduate School of Medicine, 7-5-1 Kusunoki-cho, Chuo-ku, Kobe, 650-0017 Japan; Division of Rehabilitation Medicine, Kobe University Graduate School of Medicine, 7-5-1 Kusunoki-cho, Chuo-ku, Kobe, 650-0017 Japan

## Abstract

**Introduction:**

Nutrient deprivation is a likely contributor to intervertebral disc (IVD) degeneration. Silent mating type information regulator 2 homolog 1 (SIRT1) protects cells against limited nutrition by modulation of apoptosis and autophagy. However, little evidence exists regarding the extent to which SIRT1 affects IVD cells. Therefore, we conducted an in vitro study using human IVD nucleus pulposus (NP) cells.

**Methods:**

Thirty-two IVD specimens were obtained from patients who underwent surgical intervention and were categorized based on Pfirrmann IVD degeneration grades. Cells were isolated from the NP and cultured in the presence of recombinant human SIRT1 (rhSIRT1) under different serum conditions, including 10 % (v/v) fetal bovine serum (FBS) as normal nutrition (N) and 1 % (v/v) FBS as low nutrition (LN). 3-Methyladenine (3-MA) was used to inhibit autophagy. Autophagic activity was assessed by measuring the absorbance of monodansylcadaverine and immunostaining and Western blotting for light chain 3 and p62/SQSTM1. Apoptosis and pathway analyses were performed by flow cytometry and Western blotting.

**Results:**

Cells cultured under LN conditions decreased in number and exhibited enhanced autophagy compared with the N condition. Medium supplementation with rhSIRT1 inhibited this decrease in cell number and induced an additional increase in autophagic activity (*P* < 0.05), whereas the combined use of rhSIRT1 and 3-MA resulted in drastic decreases in cell number and autophagy (*P* < 0.05). The incidence of apoptotic cell death increased under the LN condition, which was decreased by rhSIRT1 (*P* < 0.05) but increased further by a combination of rhSIRT1 and 3-MA (*P* < 0.05). Under LN conditions, NP cells showed a decrease in antiapoptotic Bcl-2 and an increase in proapoptotic Bax, cleaved caspase 3, and cleaved caspase 9, indicating apoptosis induction via the mitochondrial pathway. These changes were suppressed by rhSIRT1 but elevated further by rhSIRT1 with 3-MA, suggesting an effect of rhSIRT1-induced autophagy on apoptosis inhibition. Furthermore, the observed autophagy and apoptosis were more remarkable in cells from IVDs of Pfirrmann grade IV than in those from IVDs of Pfirrmann grade II.

**Conclusions:**

SIRT1 protects against nutrient deprivation-induced mitochondrial apoptosis through autophagy induction in human IVD NP cells, suggesting that rhSIRT1 may be a potent treatment agent for human degenerative IVD disease.

## Introduction

Lower back pain affects up to 85 % of people at some point during their lives and causes severe incapacity that increases medical expenses and affects the workforce [1]. In the United States, lower back pain results in annual health care and related costs of approximately $100 billion [2]. Although the cause of lower back pain is considered to be an interdependent multifactorial process, intervertebral disc (IVD) degeneration is one of the major pathological processes implicated [3–5].

The IVD is structurally an avascular tissue that includes an inner nucleus pulposus (NP) surrounded by an outer annulus fibrosus (AF), which is sandwiched between the cartilaginous endplates of contiguous vertebral bodies. The nutritional supply to NP cells depends on diffusion, primarily through the endplates, which is reduced by aging- and smoking-induced sclerosis of the subchondral bone [6]. A loss of nutritional supply ultimately leads to apoptotic cell death in the IVD [7, 8]. In particular, apoptosis of NP cells is considered to be the first sign of IVD degeneration [9]. Therefore, treatments targeting cell apoptosis need to be developed to inhibit or prevent degenerative IVD disease.

Silent mating type information regulator 2 homolog 1 (SIRT1), a mammalian homolog of silent information regulator 2, is a nicotinamide-dependent class 3 histone deacetylase that is essential for cell survival and increases the longevity of mammalian cells under calorie restriction [10]. In response to cellular energy loss, SIRT1 regulates multiple cellular processes, including the cell cycle, metabolism, and apoptosis [11, 12]. Previously, we hypothesized that SIRT1 might play an important role in homeostasis of IVD tissues, especially during the early phase of IVD degeneration. We subsequently revealed for the first time the expression of SIRT1 in human NP cells, which markedly increases in IVDs during early degeneration stages, thereby promoting extracellular matrix (ECM) metabolism and cell proliferation [13]. More recently, it has been shown that SIRT1 prevents human chondrocyte apoptosis, suppresses catabolic changes in gene expression, and accelerates osteoarthritis in conditional SIRT1-deficient mice [14–16]. SIRT1 has been considered to inhibit apoptosis by deacetylation of nuclear proteins such as p53 and nuclear factor κB (NF-κB) [17, 18]. Thus, it is conceivable that SIRT1 is a key regulator of mammalian musculoskeletal cell survival through modulation of apoptosis.

SIRT1 is also known to regulate the autophagy–lysosome pathway [19]. Autophagy-related genes *Atg5*, *Atg7*, and *Atg8*, which regulate autophagy, are deacetylated by SIRT1 in a nicotinamide-dependent manner, and this deacetylation is accelerated by limited nutrition [19]. Autophagy is an important cell survival mechanism activated by calorie restriction in which cells digest and recycle their own damaged proteins and organelles [12]. It has been revealed that autophagy is associated with certain degenerative diseases such as osteoarthritis [20], Parkinson’s disease [21], and Alzheimer’s disease [22]. Autophagy and apoptosis are closely associated in the pathological processes of diverse human diseases [19].

On the basis of the antiapoptotic and proautophagic roles of SIRT1, we hypothesized that SIRT1 might play a key role in the homeostasis of IVD cells under nutrient deprivation at the early phase of IVD degeneration. Furthermore, exogenous administration of recombinant human silent mating type information regulator 2 homolog 1 (rhSIRT1) protein into an IVD might contribute to cell survival by inhibition of apoptosis though autophagy induction. The purpose of this study was to elucidate the effects of exogenous SIRT1 on autophagy and apoptosis under limited nutrition in human IVD NP cells.

## Methods

### Ethics statement

The collection and use of human IVD specimens were approved by the ethics committee of Kobe University Hospital. All samples were obtained in accordance with the World Medical Association Declaration of Helsinki Ethical Principles for Medical Research Involving Human Subjects. This study was approved by the institutional review board of Kobe University Graduate School of Medicine. All patients provided written informed consent.

### Culture of human nucleus pulposus cells

Thirty human NP tissue samples were obtained from consenting patients during surgical procedures (lumbar disc herniation, 13 cases; lumbar spinal stenosis, 10 cases; idiopathic scoliosis, 9 cases). There were 18 males and 14 females with a mean age of 32.4 years (range 11–80 years). Degeneration grades of the IVDs were determined by magnetic resonance imaging and classified according to the Pfirrmann grading system [23]. The degeneration grades were represented by 9 cases of grade II IVD and 23 cases of grade IV IVD. All Pfirrmann grade II IVDs were obtained from young patients with idiopathic scoliosis (Table 1). To clearly compare early degenerative IVDs (Pfirrmann grade II) with advanced degenerative IVDs (Pfirrmann grade IV), moderate degenerative IVDs of Pfirrmann grade III were not analyzed in the present study.

**Table 1:** Human nucleus pulposus tissue sampling

	Pfirrmann grade II	Pfirrmann grade IV	*P* value
	(n = 9)	(n = 23)	
Mean age (yr)	13.6 ± 1.5	39.8 ± 11.5	<0.01
Sex (male:female)	2:7	16:7	<0.01
Diseases	Idiopathic scoliosis: 9	Lumbar disc herniation: 13	
		Lumbar spinal stenosis: 10	

The NP was carefully separated from the AF immediately after surgery. The tissue specimens were centrifuged and then subjected to sequential enzyme digestions at 37 °C with 0.025 % (w/v) collagenase P (Roche Applied Science, Indianapolis, IN, USA) and 0.001 % (w/v) deoxy-ribonuclease 2 (DNase 2) (Sigma-Aldrich, St Louis, MO, USA) for 12 h. The digested NP tissues were placed in complete tissue culture medium consisting of Dulbecco’s modified Eagle’s medium (DMEM; Sigma-Aldrich) supplemented with 10 % (v/v) heat-inactivated fetal bovine serum (FBS; Sigma-Aldrich), 100 U/ml penicillin, and 100 mg/ml streptomycin. When confluent, the NP cells were released and transferred to 35-mm culture dishes for 3 days of preculture at 37 °C in 20 % (v/v) O_2_, 5 % (v/v) CO_2_, and 1000 mg/L glucose. These isolated cells were defined as NP cells.

### Treatment

To induce nutritional stress, NP cells were cultured in DMEM supplemented with 1 % (v/v) FBS, defined as the low nutritional (LN) condition, compared with 10 % (v/v) FBS defined as the normal nutritional (N) condition. In this study, to extrapolate possible clinical applications of SIRT1 to IVDs, exogenous supplementation of rhSIRT1 (Sigma-Aldrich) and not transfection of the SIRT1-encoding gene was employed. To inhibit autophagy, we used 3-methyladenine (3-MA; Santa Cruz Biotechnology, Santa Cruz, CA, USA), a specific inhibitor of the early stages of the autophagic process.

After 3 days of preculture, the NP cells were assigned to one of four culture groups (Table 2) as follows: group N, normal nutritional (N) condition cultured with 10 % (v/v) FBS; group LN, low nutritional (LN) condition cultured with 1 % (v/v) FBS; group LN + SIRT1, low nutritional (LN) condition cultured with 1 % (v/v) FBS and rhSIRT1 (10 μM); and group LN + SIRT1 + 3-MA, low nutritional (LN) condition cultured with 1 % (v/v) FBS, rhSIRT1 (10 μM), and 3-MA (5 mM). Introduction of rhSIRT1 using a protein delivery reagent with culture medium was repeated every 3 days at the same time as media changes.

**Table 2:** Experimental groups

Culture groups	Culture conditions
Group N	DMEM with 10 % (v/v) FBS
Group LN	DMEM with 1 % (v/v) FBS
Group LN + SIRT1	DMEM with 1 % (v/v) FBS + 10 μM rhSIRT1
Group LN + SIRT1 + 3-MA	DMEM with 1 % (v/v) FBS + 10 μM rhSIRT1 + 5 mM 3-MA

*Abbreviations: DMEM* Dulbecco’s modified Eagle’s medium, *FBS* fetal bovine serum, *LN* low nutrition, *3-MA* 3-methyladenine, *N* normal nutrition, *rhSIRT1* recombinant human silent mating type information regulator 2 homolog 1, *SIRT1* silent mating type information regulator 2 homolog 1

### Delivery efficacy of rhSIRT1

rhSIRT1 was introduced into NP cells using Pro-DeliverIN™ (OZ Biosciences, Marseille, France), a protein delivery reagent, according to the manufacturer’s instructions. The delivery efficiency of Pro-DeliverIN™ in NP cells was measured using rhSIRT1 labeled with a HiLyte Fluor™ 555 Labeling Kit-NH_2_ (Dojindo Molecular Technology, Rockville, MD, USA). A microscopic visual count was performed as described previously to calculate the delivery efficiency of rhSIRT1 [13].

### Analysis of total NP cell numbers

NP cells that were approximately 60–70 % confluent were treated with 10 μM rhSIRT1 or 5 mM 3-MA under N or LN conditions in six-well plates at a density of 1.5 × 10^5^ cells/well. Briefly, after 3, 7, and 14 days of initial treatment, images of five random fields were obtained with Motic Images Plus 2.2 software (Shimadzu, Kyoto, Japan). The number of NP cells in each well was then calculated and represented as the percentage of the baseline cell number.

### Immunohistochemical staining

After 72 h of treatment, the cells were fixed in 4 % (w/v) paraformaldehyde/phosphate-buffered saline (PBS) for 15 minutes at room temperature, permeabilized, and blocked in 0.2 % (v/v) Triton X-100 in PBS for 30 minutes. To analyze autophagy, the cells were incubated with a primary antibody against human light chain 3 (LC3; Santa Cruz Biotechnology) overnight at 4 °C, followed by an Alexa Fluor 594–conjugated secondary antibody (Invitrogen, Carlsbad, CA, USA) for 30 minutes at room temperature. Images were obtained using a BZ-9000 microscope (Keyence, Osaka, Japan).

### Analysis of autophagy activity

Autophagy activity was assessed by using an autophagy staining kit (Cayman Chemical, Ann Arbor, MI, USA). The absorbance of monodansylcadaverine (MDC), which enters multilamellar bodies by both an ion-trapping mechanism and interactions with membrane lipids as a probe to detect autophagic vacuoles, was measured using a microplate reader after 6, 12, 24, 48, and 72 h of treatment. Autophagy activity was calculated as the MDC absorbance normalized to the cell number.

### Analysis of apoptosis

Apoptosis was assessed using a MEBCYTO apoptosis kit (MBL International, Nagoya, Japan) and flow cytometry. Early apoptotic cells stained only with annexin V conjugated with fluorescein isothiocyanate (annexin V-FITC), and late apoptotic/necrotic cells stained with propidium iodide (PI) as well as annexin V-FITC. After 3, 7, and 14 days of treatment, treated NP cells were isolated, centrifuged, and then incubated in 100 μl binding buffer containing annexin V-FITC and PI. Apoptotic cells, indicated by positive staining for annexin V-FITC only or annexin V-FITC and PI, were counted by flow cytometry and represented as the percentage of the total cell number.

### Sodium dodecyl sulfate-polyacrylamide gel electrophoresis and Western blotting

Treated NP cells were lysed on ice for 30 minutes in lysis buffer containing protease and phosphatase inhibitors (Roche Diagnostics, Basel, Switzerland). Total cellular proteins were centrifuged and collected. The separated proteins were transblotted electrically onto a blotting membrane (Amersham Biosciences, Arlington Heights, IL, USA). The membranes were probed with primary antibodies followed by a horseradish peroxidase–conjugated secondary antibody. Proteins were visualized with ECL Plus reagent (Amersham Biosciences) and a Chemilumino analyzer LAS-3000 mini (Fujifilm, Tokyo, Japan). The expression of acetylated and total p53, as well as of acetylated and total NF-κB proteins, was detected to elucidate the deacetylation potential of SIRT1. LC3 and p62 protein/sequestome 1 (p62/SQSTM1) were detected to monitor autophagy. In addition, Bcl-2-associated X protein (Bax), B-cell lymphoma (Bcl-2), caspase 3, and caspase 9 proteins were analyzed to determine the involved pathways of SIRT1 during apoptosis. The intensities of the bands were quantified using ImageJ analysis software, version 1.45 (NIH Image; National Institutes of Health, Bethesda, MD, USA). The antibodies used in this study were as follows: rabbit monoclonal or polyclonal antibodies against acetylated and total p53, acetylated and total NF-κB, LC3, p62/SQSTM1, Bax, Bcl-2, caspases 3 and 9, and cleaved caspases 3 and 9 (Cell Signaling Technology, Danvers, MA, USA); a mouse-monoclonal antibody against α-tubulin (Sigma-Aldrich); and horseradish peroxidase–conjugated anti-rabbit and anti-mouse antibodies (Amersham Biosciences).

### Statistical analysis

Data are expressed as the mean ± standard deviation. Three-way analysis of variance with the Tukey–Kramer post hoc test was used to assess differences in experimental groups. Student’s *t* test was used to evaluate differences between the Pfirrmann grades II and IV groups. *P* values <0.05 were regarded as statistically significant. Statistical analyses were performed using PASW Statistics 18 (IBM SPSS, Armonk, NY, USA).

## Results

### Introduction of recombinant human SIRT1 into nucleus pulposus cells under low nutritional conditions maintained deacetylation potential

**Fig. 1:**
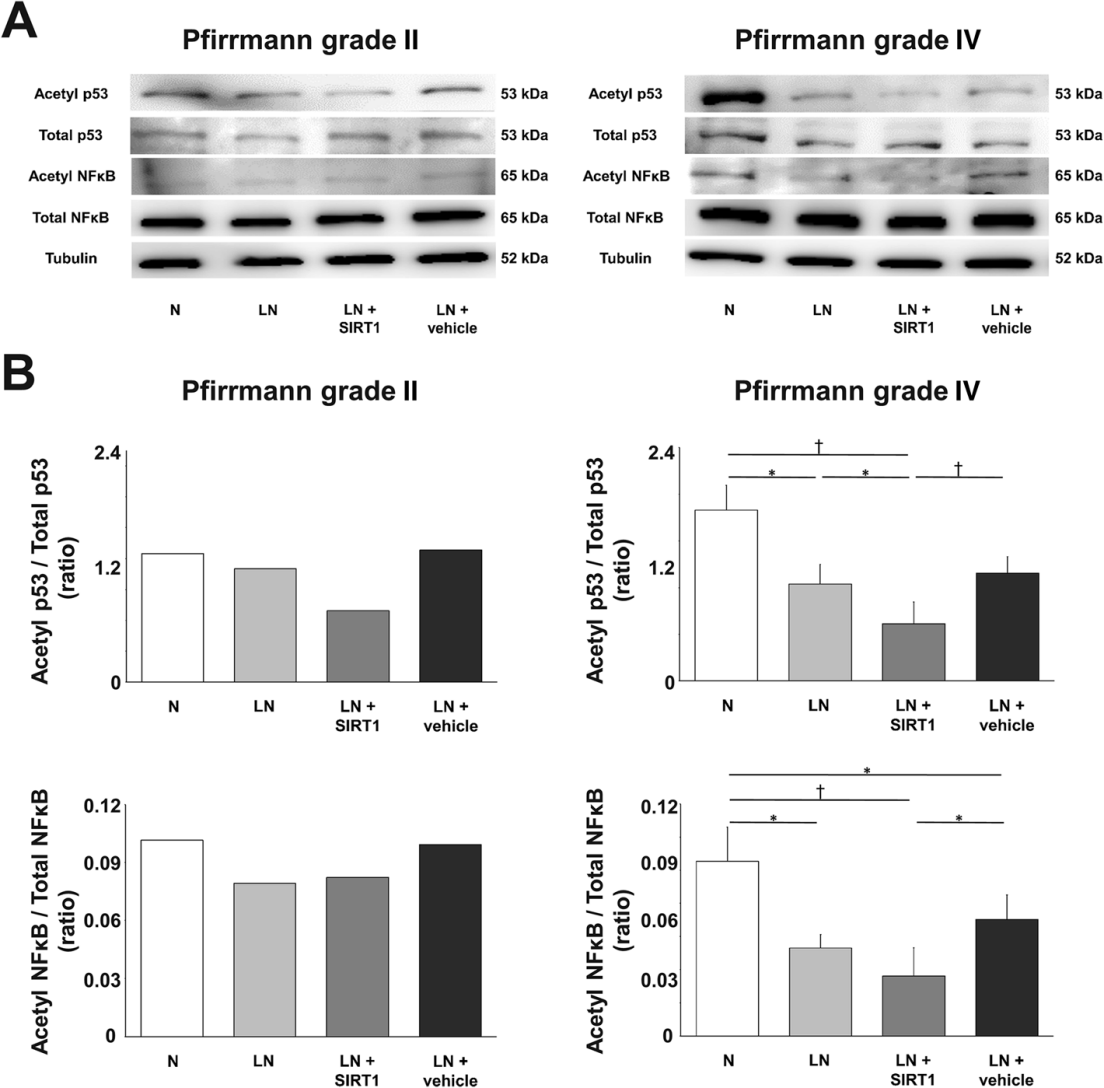
Deacetylation potential of recombinant human silent mating type information regulator 2 homolog 1 (rhSIRT1) introduced into human nucleus pulposus cells. **a** Western blot analysis of acetylated and total p53, and acetylated and total nuclear factor κB (NF-κB) in nucleus pulposus cells from intervertebral discs (IVDs) of Pfirrmann grades II and IV after 72 h of treatment with 10 μM rhSIRT1 under normal nutrition (N) conditions with 10 % (v/v) fetal bovine serum (FBS) or low nutrition (LN) conditions with 1 % (v/v) FBS. Cells introduced with a nonspecific protein, R-phycoerythrin, were used as the control. α-Tubulin was used as the loading control. **b** Ratio of acetylated p53 to total p53 and acetylated NF-κB to total NF-κB. Data are expressed as the mean ± standard deviation (n = 1 for cells from grade II IVD and n = 4 for cells from grade IV IVDs). *P < 0.05; ^†^ *P* < 0.01, three-way analysis of variance with the Tukey–Kramer post hoc test

The delivery efficiency of 10 μM rhSIRT1 using protein delivery reagent was 37.9 % in our previous study [13]. In the Pfirrmann grade II IVD (n = 1), introduction of rhSIRT1 reduced acetylated p53 but did not reduce acetylated NF-κB levels. In Pfirrmann grade IV IVDs (n = 4), introduction of rhSIRT1 reduced both acetylated p53 and acetylated NF-κB levels. This acetylation potential of rhSIRT1 was more prominent in Pfirrmann grade IV IVDs than in the Pfirrmann grade II IVDs. These findings indicate the deacetylation potential of rhSIRT1 under LN conditions (Fig. 1a, b).

### Recombinant human SIRT1 inhibited the decrease in nucleus pulposus cell number under low nutritional conditions

**Fig. 2:**
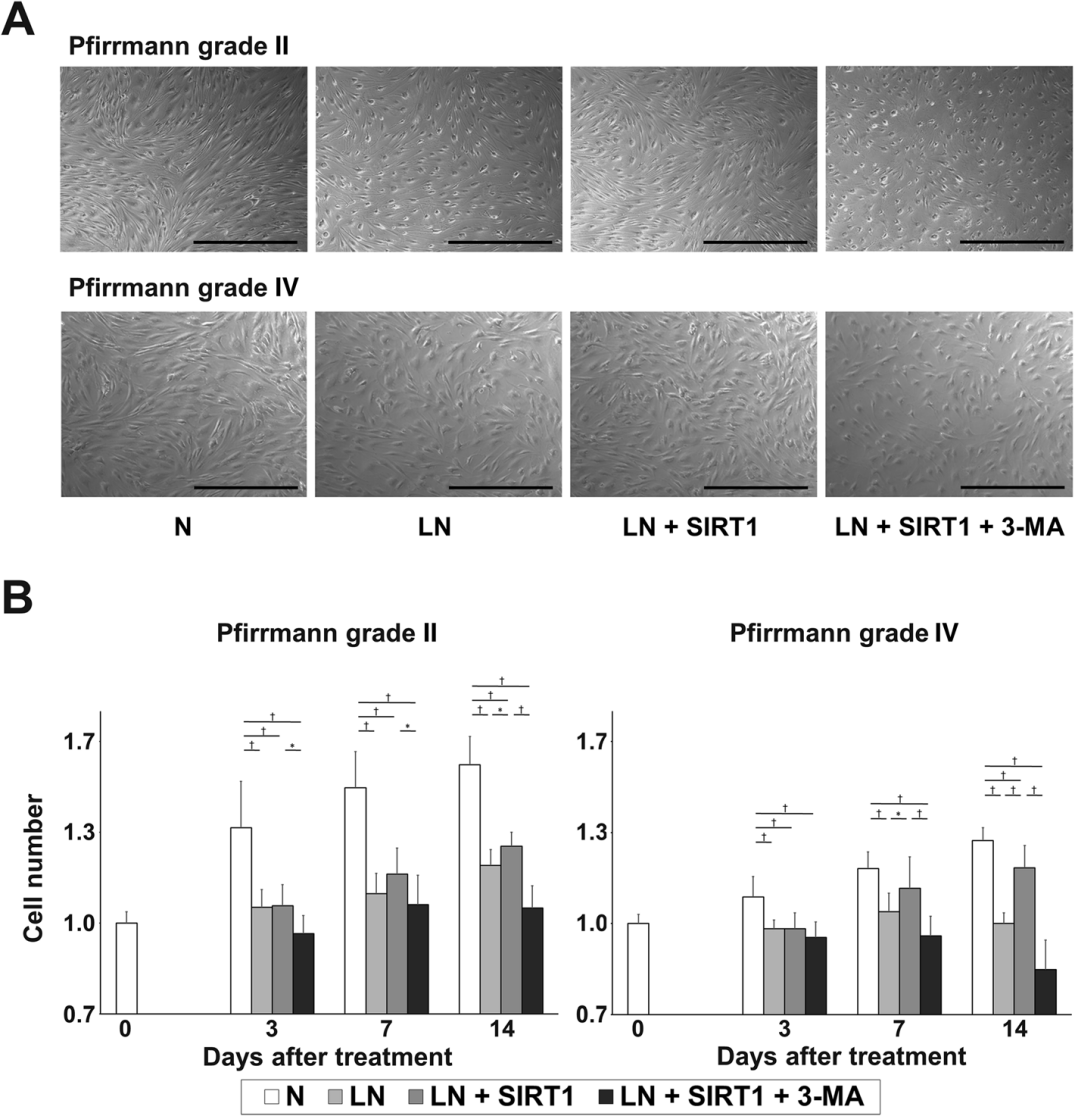
Effect of recombinant human silent mating type information regulator 2 homolog 1 (rhSIRT1) on the number of human nucleus pulposus (NP) cells. **a** Morphological appearance of NP cells from intervertebral discs (IVDs) of Pfirrmann grade II and IV after 14 days of treatment with 10 μM rhSIRT1 or 5 mM 3-methyladenine (3-MA) under normal nutrition (N) conditions with 10 % (v/v) fetal bovine serum (FBS) or low nutrition (LN) conditions with 1 % (v/v) FBS (bars, 250 μm). **b** Changes in the number of NP cells from IVDs of Pfirrmann grades II and IV after 0, 3, 7, and 14 days of treatment. Data are expressed as the mean ± standard deviation (n = 5). *P < 0.05; ^†^ *P* < 0.01, three-way analysis of variance with the Tukey–Kramer post hoc test

In this experiment, morphological changes were not observed in any experimental group (Fig. 2a).

In Pfirrmann grade II IVDs (n = 5), the total number of NP cells in group N showed a time-dependent increase over 14 days (day 14 + 52.2 % vs. day 0). NP cells were treated with each condition in six-well plates at a relatively high density of IVD cells (approximately 50–60 % confluent). NP cells did not double, even after 14 days of culture in 10 % (v/v) serum-supplemented media. This result suggests the very slow cell proliferation of human NP cells or reduced cell division due to the confluence in our cell culture system. The total number of NP cells in group LN + SIRT1 showed a slight increase and was significantly higher than that in group LN at only day 14 (*P* < 0.05). In addition, the total NP cell number in group LN + SIRT1 + 3-MA was significantly lower than that in group LN + SIRT1 at every time point (*P* < 0.05) (day 14, group LN: −22 %, group LN + SIRT1: −18 %, group LN + SIRT1 + 3-MA: −32 % vs. Group N) (Fig. 2b). In Pfirrmann grade IV IVDs (n = 5), the number of NP cells in group N showed a time-dependent increase (day 14 + 27.3 % vs. day 0). Cell numbers in group LN were significantly lower than those in group N at every time point (*P* < 0.01). The number of NP cells in group LN + SIRT1 showed a time-dependent increase with a significant difference compared with that in group LN at days 7 and 14. However, NP cell number in group LN + SIRT1 + 3-MA was significantly lower than that in group LN + SIRT1 at days 7 and 14 (*P* < 0.05) (day 14, group LN: −22 %, group LN + SIRT1: −8 %, group LN + SIRT1 + 3-MA: −34 % vs. Group N) (Fig. 2b).

### Recombinant human SIRT1 accelerated autophagy activity under low nutrition conditions

**Fig. 3:**
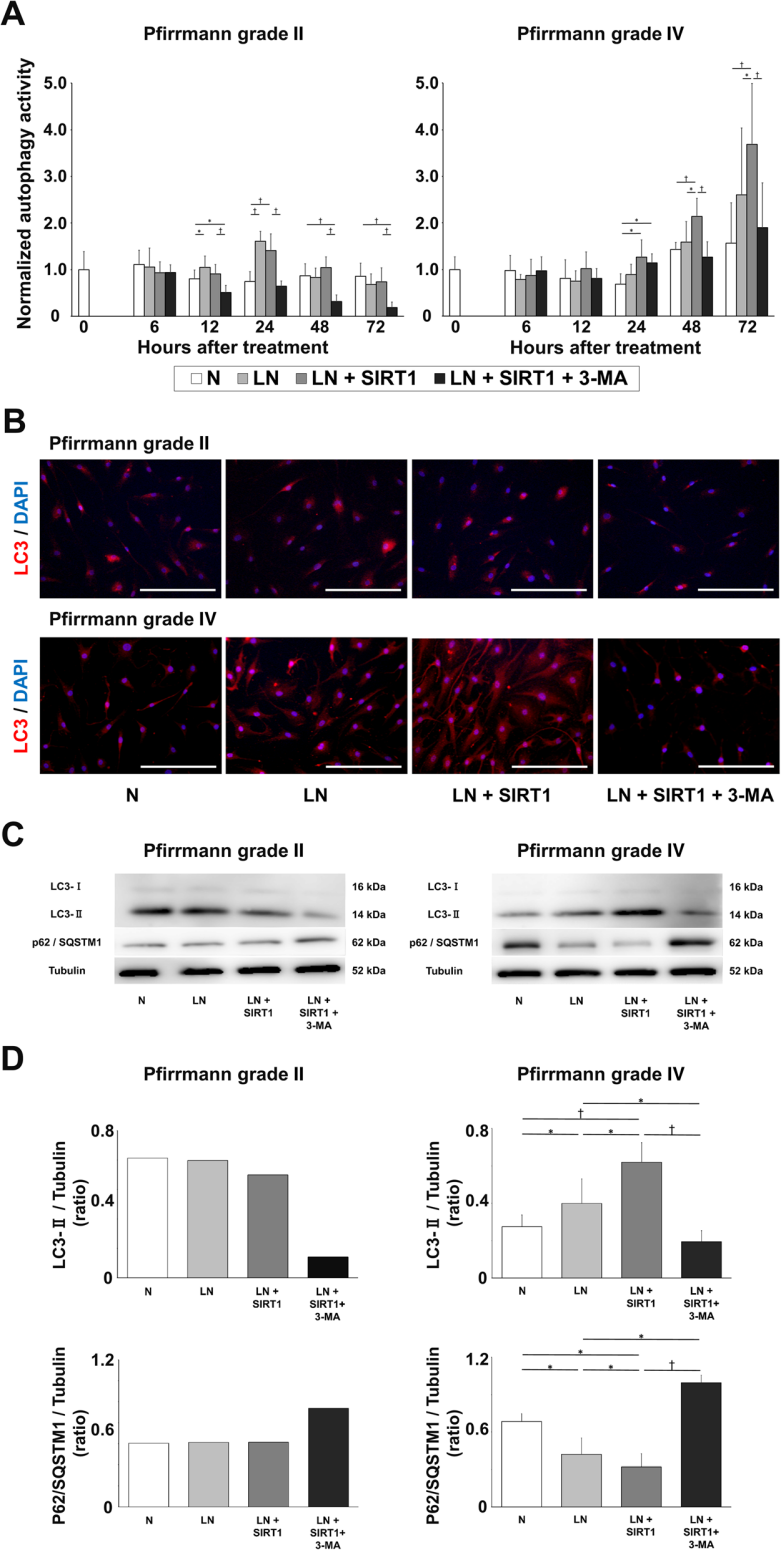
Effect of recombinant human silent mating type information regulator 2 homolog 1 (rhSIRT1) on the level of autophagy in human nucleus pulposus (NP) cells. **a** Changes in the normalized autophagy activity of NP cells from intervertebral discs (IVDs) of Pfirrmann grades II and IV after 0, 6, 12, 24, 48, and 72 h of treatment with 10 μM rhSIRT1 or 5 mM 3-methyladenine (3-MA) under normal nutrition (N) conditions with 10 % (v/v) fetal bovine serum (FBS) or low nutrition (LN) conditions with 1 % (v/v) FBS. Autophagy activity was measured by the absorbance of monodansylcadaverine and normalized to the cell number. Data are expressed as the mean ± standard deviation (n = 5). *P < 0.05; ^†^ *P* < 0.01, three-way analysis of variance with the Tukey–Kramer post hoc test. **b** Immunofluorescence images of light chain 3 (LC3) in NP cells from IVDs of Pfirrmann grades II and IV after 72 h of treatment. 4′,6-Diamino-2-phenylindole (DAPI) was used for counterstaining. *Red fluorescence* indicates LC3, and *blue fluorescence* indicates nuclei (bars, 100 μm). **c** Western blot analysis of LC3 and p62/SQSTM1 in NP cells from IVDs of Pfirrmann grades II and IV after 72 h of treatment. α-Tubulin was used as the loading control. **d** Ratio of LC3-II to tubulin and p62 protein/sequestome 1 (p62/SQSTM1) to tubulin. Data are expressed as the mean ± standard deviation (n = 1 for cells from the grade II IVD and n = 3 for cells from grade IV IVDs). *P < 0.05; ^†^ *P* < 0.01, three-way analysis of variance with the Tukey–Kramer post hoc test

First, autophagy was quantitatively evaluated by the absorbance of MDC. In Pfirrmann grade II IVDs (n = 5), the normalized autophagy activity of NP cells in group N showed no significant alteration for 72 h. Autophagy activity in groups LN and LN + SIRT1 was significantly higher than that in group N at 24 h (*P* < 0.01). However, there was no significant difference in normalized autophagy activity between groups LN and LN + SIRT1. Autophagy activity in group LN + SIRT1 + 3-MA was significantly lower than that in group LN + SIRT after 12 h (*P* < 0.01) (24 h, group LN: +115 %, group LN + SIRT1: +88 %, group LN + SIRT1 + 3-MA: −14 % vs. group N) (Fig. 3a). In Pfirrmann grade IV IVDs (n = 5), the normalized autophagy activity in group N showed no significant alteration for 72 h. Autophagy activity in group LN was higher than that in group N after 24 h. Moreover, autophagy activity of NP cells was significantly stimulated in group LN + SIRT1 compared with group LN after 48 h (*P* < 0.05). However, autophagy activity in group LN + SIRT1 + 3-MA was significantly lower than that in group LN + SIRT1 after 48 h (*P* < 0.01) (72 h, group LN: +66 %, group LN + SIRT1: +136 %, group LN + SIRT1 + 3-MA: +21 % vs. group N) (Fig. 3a).

Next, immunofluorescence analysis for LC3 was performed at 72 h after treatment. In Pfirrmann grade II IVDs, immunoreactivity for LC3 was measured in group N, as the basal level, which showed little difference among groups N, LN, and LN + SIRT1. In Pfirrmann grade IV IVDs, LC3 immunoreactivity of group LN was higher than that of group N. Furthermore, LC3 immunoreactivity in group LN + SIRT1 was higher than that in groups N and LN. However, in group LN + SIRT1 + 3-MA, LC3 immunoreactivity was attenuated, together with a decrease in the number of NP cells (Fig. 3b).

Finally, we examined autophagic flux by monitoring LC3 and p62/SQSTM1 at 72 h after treatment. LC3-I expression levels were quite low, making it difficult to measure the ratio of LC3-II/LC3-I. Therefore, we measured the expression level of LC3-II compared with that of tubulin. In the Pfirrmann grade II IVD (n = 1), Western blot analysis revealed the SIRT1 treatment did not affect protein expression. In Pfirrmann grade IV IVDs (n = 3), a significant increase in the amount of LC3-II was detected in group LN + SIRT1 compared with group LN and LN + SIRT1 + 3MA (*P* < 0.05). In addition, the amount of p62/SQSTM1 in group LN + SIRT1 was significantly decreased compared with the other groups (*P* < 0.05) (Fig. 3c, d).

### Recombinant human SIRT1 attenuated apoptosis under the low nutrition conditions

**Fig. 4:**
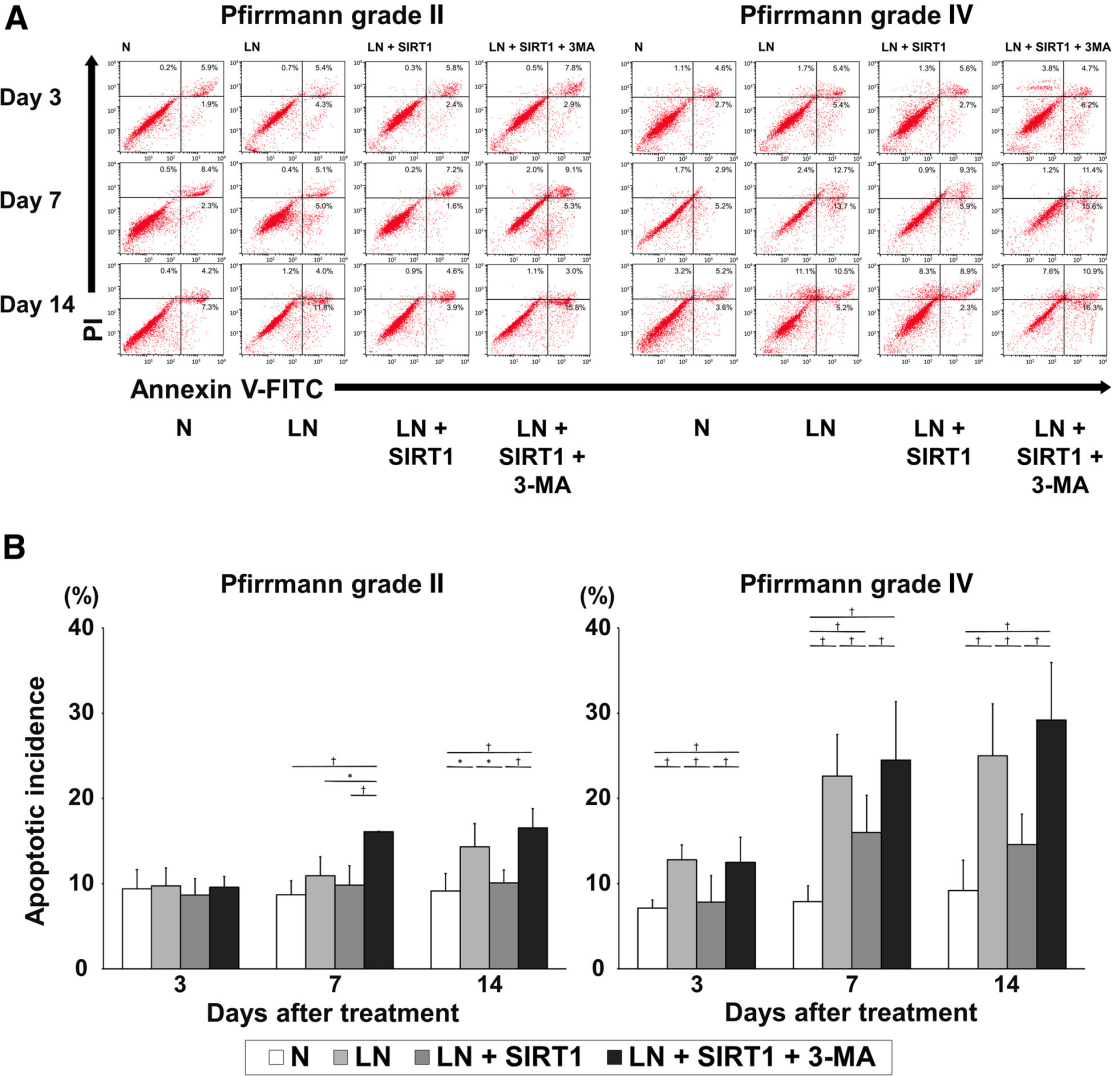
Effect of recombinant human silent mating type information regulator 2 homolog 1 (rhSIRT1) on apoptosis of human nucleus pulposus cells. **a** Flow cytometric analysis of annexin V conjugated with fluorescein isothiocyanate (Annexin V-FITC) and propidium iodide–stained nucleus pulposus cells from intervertebral discs (IVDs) of Pfirrmann grades II and IV after 3, 7, and 14 days of treatment with 10 μM rhSIRT1 or 5 mM 3-methyladenine (3-MA) under normal nutrition (N) conditions with 10 % (v/v) fetal bovine serum (FBS) or low nutrition (LN) conditions with 1 % (v/v) FBS. **b** Changes in the percentage of apoptotic NP cells from IVDs of Pfirrmann grades II and IV after 3, 7, and 14 days of treatment. Data are expressed as the mean ± standard deviation (n = 5 for cells from grade II IVDs and n = 8 for cells from grade IV IVDs). *P < 0.05; ^†^ *P* < 0.01, three-way analysis of variance with the Tukey–Kramer post hoc test

In Pfirrmann grade II IVDs (n = 5), apoptosis in group N, as detected by flow cytometry, was not altered for 14 days. Apoptosis in group LN + SIRT1 was also unaltered for 14 days. However, the extent of apoptosis in group LN + SIRT1 + 3-MA was significantly increased compared with that of group LN + SIRT1 at days 7 and 14 (*P* < 0.01) (day 14, group N: 9 %, group LN: 14 %, group LN + SIRT1: 10 %, group LN + SIRT1 + 3-MA: 16 %). In Pfirrmann grade IV IVDs (n = 8), the extent of apoptosis in group LN, LN + SIRT1, and LN + SIRT1 + 3-MA was higher than that in Pfirrmann grade II IVDs. Apoptosis in group N was maintained at the initial level at day 3. In group LN, apoptosis increased in a time-dependent manner compared with group N at every time point (*P* < 0.01). The apoptotic incidence of group LN + SIRT1 was also increased compared with that of group N at day 7 (*P* < 0.01), but it was significantly lower than that of group LN at every time point (*P* < 0.05). Group LN + SIRT1 + 3-MA demonstrated the highest levels of apoptosis among all groups (*P* < 0.01) (day 14, group N: 9 %, group LN: 24 %, group LN + SIRT1: 14 %, group LN + SIRT1 + 3-MA: 29 %) (Fig. 4a, b). On the basis of our results, basal levels of apoptosis in group N were not so high. This may indicate physiological cell life turnover or programmed cell death. Treatment with rhSIRT1 can suppress limited nutrition-induced non-physiological apoptosis, but it may not affect physiological apoptosis.

### Recombinant human SIRT1 modulated the mitochondrial apoptotic pathway under low nutrition conditions

**Fig. 5:**
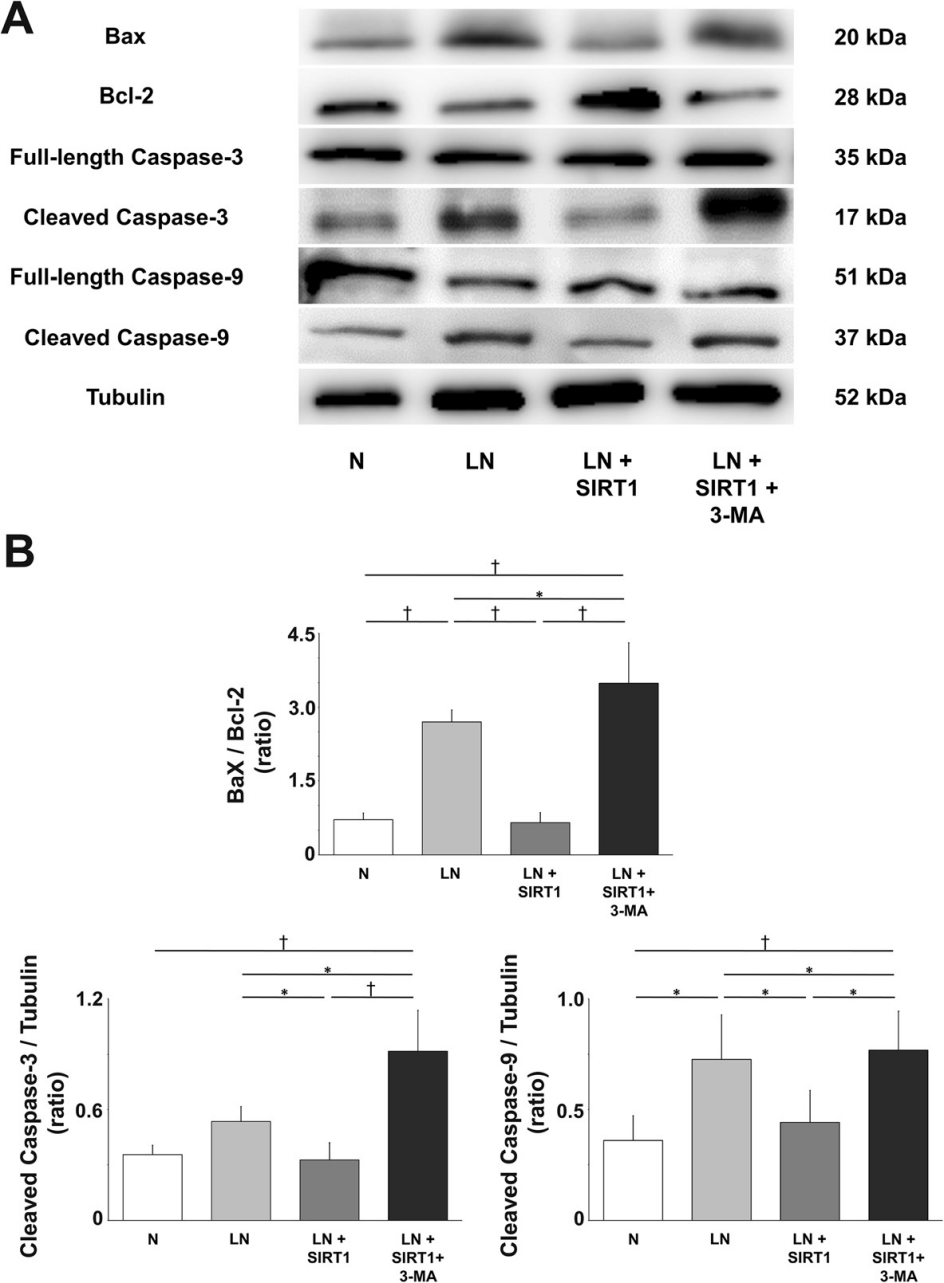
Effect of recombinant human silent mating type information regulator 2 homolog 1 (rhSIRT1) on apoptosis mediated through the mitochondrial pathway in human nucleus pulposus cells. **a** Western blot analysis of Bcl-2-associated X protein (Bax), Bcl-2, full-length caspases 3 and 9, and cleaved caspases 3 and 9 in nucleus pulposus cells from intervertebral discs of Pfirrmann grade IV after 72 h of treatment with 10 μM rhSIRT1 or 5 mM 3-methyladenine (3-MA) under normal nutrition (N) conditions with 10 % (v/v) fetal bovine serum (FBS) or low nutrition (LN) conditions with 1 % (v/v) FBS. α-Tubulin was used as a loading control. **b** Ratio of Bax to Bcl-2 and cleaved caspases 3 and 9 to tubulin. Data are expressed as the mean ± standard deviation (n = 4). *P < 0.05; ^†^ *P* < 0.01, three-way analysis of variance with the Tukey–Kramer post hoc test

Apoptotic pathway proteins were analyzed in Pfirrmann grade IV IVDs (n = 4) by Western blotting. Higher amounts of both cleaved caspases 3 and 9 were detected in group LN compared with group N. In addition, Bax protein expression was increased and Bcl-2 was attenuated in group LN. However, this enhancement of both cleaved caspases 3 and 9 protein expression in group LN was counteracted in group LN + SIRT1. Moreover, Bax expression was attenuated and Bcl-2 was increased in group LN + SIRT1. This observation shows that rhSIRT1 has an antiapoptotic effect mediated through the mitochondrial pathway under LN conditions. Furthermore, when autophagy was inhibited by 3-MA, rhSIRT1-induced antiapoptosis was attenuated in group LN + SIRT1 + 3-MA (Fig. 5a, b).

## Discussion

In the present study, we demonstrate that reduced nutrition induced apoptosis through the mitochondrial pathway in human IVD NP cells and that rhSIRT1 prevented this apoptosis as well as induced autophagy. Pharmacological inhibition of autophagy resulted in a marked increase in apoptosis, suggesting an antiapoptotic effect of rhSIRT1 through induction of autophagy. Furthermore, these effects of rhSIRT1 on autophagy and apoptosis in human NP cells were more prominent in Pfirrmann grade IV IVDs than in Pfirrmann grade II IVDs.

Published evidence suggests that excessive apoptosis of IVD cells is a potential cause of IVD degeneration [3, 5, 24, 25]. Sudo et al. demonstrated that serum starvation and annular needle puncture models induce apoptosis leading to IVD degeneration, and they also revealed that both Bcl-2 overexpression and caspase 3 knockdown prevent apoptosis and degenerative alterations [26, 27]. We also found that apoptosis of both AF and NP cells is mediated through the mitochondrial pathway in a rat tail static compression model of disc degeneration [28]. In surgically obtained herniated IVDs, Park et al. revealed that NP cell apoptosis is also induced by the mitochondrial pathway [9]. Consequently, prevention of apoptosis in NP cells is considered to be a therapeutic strategy for IVD regeneration. In the present study, reduced serum concentrations induced apoptosis in both early and advanced degenerative IVDs. This proapoptotic effect induced by nutritional stress was more remarkable in advanced degenerative IVDs than in early degenerative IVDs. On the basis of these results, advanced degenerative NP cells are supposed to be more sensitive to nutritional stress than early degenerative NP cells, leading to apoptosis more readily.

Researchers in several studies have reported that prevention of apoptosis by platelet-derived growth factor [29], Bcl-2 [27], caspase 3 small interfering RNA (siRNA) [26], or a caspase inhibitor [30] has positive effects on ECM metabolism in IVD cells. In the current study, introduced rhSITR1 reduced apoptosis under LN conditions in human NP cells from Pfirrmann grades II and IV IVDs. Furthermore, this antiapoptotic effect of rhSIRT1 was attenuated by 3-MA, an inhibitor of autophagy. Therefore, introduced rhSIRT1 prevented apoptosis through autophagy induction. In addition, apoptosis inhibition was more obvious in Pfirrmann grade IV IVDs than in Pfirrmann grade II IVDs. We also found that introduced rhSIRT1 reduced acetylation of p53, decreased proapoptotic Bax expression, apoptotic cleavage of caspases 3 and 9, and increased antiapoptotic Bcl-2 expression in human NP cells. It has been shown that deacetylation of p53 by SIRT1 contributes to inhibition of p53-dependent mitochondrial apoptosis [18, 31]. Thus, introduced rhSIRT1 potentially contributes to NP cell survival by modulation of the mitochondrial apoptotic pathway.

Autophagy is one type of cytoprotective process and ameliorates various types of cell stress. Activation of autophagy improves cell survival, whereas inhibition of autophagy promotes cell death [32]. Autophagy is known to be activated by a variety of stress stimuli, including calorie restriction [33], hypoxia [34], and DNA damage [35]. In addition, SIRT1 serves to activate autophagy under limited nutrition [19]. In the present study, introduced rhSIRT1 activated autophagy in NP cells under nutrient starvation and increased the number of NP cells through suppression of apoptosis that was inhibited by autophagy, which is in line with previous evidence.

Mounting evidence has indicated possible crosstalk between autophagy and apoptosis pathways. The interplay between autophagy and apoptosis may be important throughout development and homeostasis [36, 37]. Inhibition of autophagy by 3-MA or siRNA targeting genes related to autophagy triggers apoptosis in human HeLa cells [33]. Shen et al. reported activation of autophagy in IVDs by interleukin-1β in rat AF cells under serum deprivation. Upon inhibition of autophagy by 3-MA, apoptosis was increased in AF cells. They concluded that autophagy might protect against apoptosis of AF cells as well as against IVD degeneration [38]. The results of the present study demonstrate that autophagy, which was activated by rhSIRT1, inhibited apoptosis, leading to increases in the number of human NP cells. Notably, this effect of autophagy was more prominent in advanced degenerative IVDs. These findings suggest that autophagy plays an important role in the IVD degeneration process.

Distinct apoptotic and autophagic responses to rhSIRT1 in NP cells from Pfirrmann grade IV IVDs, compared with those from Pfirrmann grade II IVDs, may be due to different disease types and degeneration severity. We have previously reported that SIRT1 mRNA expression is higher in Pfirrmann grade III IVDs than in Pfirrmann grades II and IV IVDs [13]. We considered that SIRT1 expression might be induced in the early stage of IVD degeneration when the internal environment of the IVD begins to deteriorate because of disturbances in the nutrient supply and that it then decrease in the advanced stage of IVD degeneration. The role of SIRT1 was altered according to IVD degeneration severity. The results, which show that the sensitivity to rhSIRT1 was different between Pfirrmann grades II and IV IVDs, indicate the safety and effectiveness of rhSIRT1 as a therapeutic agent for the treatment of degenerative IVD disease.

The present study may be limited regarding the type of cells employed in experiments. We used only NP cells in this study, but both NP and AF cells are involved in the mechanisms of IVD degeneration. Moreover, the experiments were conducted using monolayer cultures with 1000 mg/L glucose and 10 % (v/v) FBS, 20 % (v/v) oxygen, and a neutral pH. However, in human IVDs, the central NP is hypoxic and the oxygen concentration is <1 % (v/v) [8]. Therefore, our culture conditions do not reflect the in vivo situation. It is now evident that nutrients, oxygen levels, and pH are powerful regulators of cellular activity and gene expression in IVDs, which can strongly influence the rates of ECM synthesis and degradation, and even cell viability [7, 39, 40]. This is a limitation of the present study. In addition, we chose a low serum environment of 1 % (v/v) FBS supplementation as a condition of nutrient deprivation because serum interacts with other nutrients and plays a major role in IVD cells [40]. Serum withdrawal to 1 % (v/v) FBS supplementation has been used as a positive apoptosis control for human AF cells in vitro and resulted in a significantly greater apoptosis incidence compared with the 20 % (v/v) FBS negative control [29]. However, it remains to be elucidated whether this serum concentration is an acceptably low nutritional condition for human IVD cells. Therefore, the effects of rhSIRT1 should be confirmed in vivo. In addition, SIRT1 is widely involved in the regulation of cellular processes [11, 12]. SIRT1 may also be directly involved in cell proliferation, as shown by the increases in cell number, or it may affect apoptosis through pathways other than autophagy. Further investigations need to be conducted.

## Conclusions

We demonstrate that limited nutrition induced apoptosis through the mitochondrial pathway and that rhSIRT1 prevented apoptosis of human NP cells through acceleration of autophagy. Furthermore, the effects of rhSIRT1 on autophagy and apoptosis in human NP cells were stronger in Pfirrmann grade IV IVDs than in Pfirrmann grade II IVDs. The actions of rhSIRT1 in human NP cells depend on the degeneration grade of the IVD. SIRT1 may be a potential candidate as a novel treatment agent for degenerative IVD disease.

## Abbreviations

AF: annulus fibrosus
Annexin V-FITC: annexin V conjugated with fluorescein isothiocyanate
*Atg*: autophagy-related gene
Bax: Bcl-2-associated X protein
Bcl-2: B-cell lymphoma
DMEM: Dulbecco’s modified Eagle’s medium
ECM: extracellular matrix
FBS: fetal bovine serum
IVD: intervertebral disc
LC3: light chain 3
LN: low nutrition
3-MA: 3-methyladenine
MDC: monodansylcadaverine
N: normal nutrition
NF-κB: nuclear factor κB
NP: nucleus pulposus
PBS: phosphate-buffered saline
PI: propidium iodide
rhSIRT1: recombinant human silent mating type information regulator 2 homolog 1
siRNA: small interfering RNA
SIRT1: silent mating type information regulator 2 homolog 1
SQSTM1: sequestome 1
